# The Interactions Between *Candida albicans* and Mucosal Immunity

**DOI:** 10.3389/fmicb.2021.652725

**Published:** 2021-06-21

**Authors:** Yujie Zhou, Lei Cheng, Yu L. Lei, Biao Ren, Xuedong Zhou

**Affiliations:** ^1^State Key Laboratory of Oral Diseases, West China Hospital of Stomatology, National Clinical Research Center for Oral Diseases, Sichuan University, Chengdu, China; ^2^Guanghua School of Stomatology, Hospital of Stomatology, Sun Yat-sen University, Guangzhou, China; ^3^Department of Periodontics and Oral Medicine, University of Michigan School of Dentistry, University of Michigan Rogel Cancer Center, University of Michigan, Ann Arbor, MI, United States

**Keywords:** *Candida albicans*, mucosa, immune cell, immune recognition, immune escape

## Abstract

Mucosa protects the body against external pathogen invasion. However, pathogen colonies on the mucosa can invade the mucosa when the immunosurveillance is compromised, causing mucosal infection and subsequent diseases. Therefore, it is necessary to timely and effectively monitor and control pathogenic microorganisms through mucosal immunity. *Candida albicans* is the most prevalent fungi on the mucosa. The *C. albicans* colonies proliferate and increase their virulence, causing severe infectious diseases and even death, especially in immunocompromised patients. The normal host mucosal immune defense inhibits pathogenic *C. albicans* through stepwise processes, such as pathogen recognition, cytokine production, and immune cell phagocytosis. Herein, the current advances in the interactions between *C. albicans* and host mucosal immune defenses have been summarized to improve understanding on the immune mechanisms against fungal infections.

## Introduction

The mucosa serves as the first line of defense against external stimuli, such as toxins, cytokines, and pathogens (Awad et al., 2017). The mucosa is typically warm and humid, making it an ideal environment for micro-organism colonization and survival, including bacteria, fungi, and viruses (Hillman et al., 2017). Notably, the dynamic balance between the mucosa and microorganisms is essential for the health of the host (Grice and Segre, 2011). Some conditional microbes can transform from common ecological to the pathogenic state depending on the condition of the body and immune function (Belkaid and Hand, 2014). The mucosal immunity monitors and regulates microbe balance to inhibit and control the occurrence of infections (Hooper et al., 2012; Belkaid and Hand, 2014).

Fungi, mainly *Candida*, can commensally colonize the mucosal surface (Williams and Lewis, 2011; Kühbacher et al., 2017). Several *Candida* species colonize the mucosa, including *C. albicans*, *Candida glabrata*, *Candida tropicalis*, *Cryptococcus neoformans*, *Candida krusei*, etc. However, *C. albicans* is the most prevalent fungus (Pincus et al., 2007). As a conditional pathogen, *C. albicans* acts as a symbiotic fungus when immunity is normal and transforms into the pathogenic state when an immune disorder occurs. Besides, *C. albicans* can create ideal survival and colonization conditions for other bacteria, with such coinfections leading to more severe infectious diseases and drug resistance (Arvanitis and Mylonakis, 2015; Krüger et al., 2019). Blood infections caused by a combination of *C. albicans* and *Staphylococcus* have a high mortality rate (Kong et al., 2016; Carolus et al., 2019). The detection rate of *C. albicans* in the oral cavity is associated with pneumonia severity, especially ventilator-associated pneumonia (VAP). Therefore, maintaining good oral health can decrease the ICU pneumonia rate (Souza et al., 2017). Polysaccharide secretion from *C. albicans* causes the development of *Streptococcus mutans* biofilms in the mouth, thus increasing dental caries (Khoury et al., 2020). The biofilms combined with *C. albicans* and *Streptococcus gordonii* have high resistance to antibacterial and antifungal antibiotics (Montelongo-Jauregui et al., 2019). A study showed that infections caused by *Staphylococcus aureus* combined with *C. albicans* are highly resistant to antibiotics (Peters and Noverr, 2013).

*C. albicans* exist in yeast form on the mucosal surface in its symbiotic state and as a hypha in a pathogenic state. Therefore, *C. albicans* hypha is the main pathogenic virulence factor that invades the host, causing local mucosal infection (Williams and Lewis, 2011). *C. albicans* mainly causes candidal leukoplakia, redness, and swelling of the mucosa in the oral cavity (Patil et al., 2015). *C. albicans* can also cause oral denture stomatitis if the host wears dentures for a long time (Gleiznys et al., 2015). *C. albicans* causes median rhomboid glossitis of the tongue if the host has smoking habits (Bihari et al., 2014). Furthermore, the gastrointestinal mucosa, has numerous *C. albicans* colonies, which can be an important reserve pool for infection to spread in the human body (Kaufman and Fairchild, 2004). It can also cause candidemia through blood-borne diffusion in severe cases (Iliev et al., 2012; Patricio et al., 2019). *C. albicans* can also form a biofilm on the human mucosa surface, reducing drug and host immune system efficacies (Nobile and Johnson, 2015).

The human mucosal immunity protects the body at the initial stage through various monitoring and defense pathways, including the initial recognition and response, activation of appropriate immune defense responses, thus limiting fungal infections. This article summarizes the current advances in the interaction mechanisms between host mucosal immunity and *C. albicans* to understand the immune response to fungal infections better. This study also provides possible antifungal targets against *C. albicans* infection.

## Recognition of Various Cellular Components of *C. albicans* by Host Immunity

**FIGURE 1:**
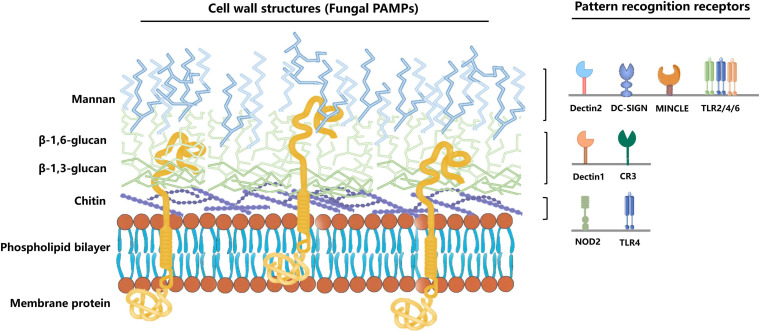
Immune cells recognize different *C. albicans* PAMPs. *C. albicans* pathogen-associated molecular patterns (PAMPs) bind to different pathogen-recognition receptors (PRRs) of the host cell to induce immunity via the fungal cell. The outer cell wall layer is mainly composed of C-linked glycoproteins, such as mannan (80–90%). The inner cell wall contains highly immunogenic chitin, β-1,3-glucan, and β-1,6-glucan. Mannan can be recognized by the Dectin-2, DC-SIGN, MINCLE, and TLR2/4/6. Besides, β-glucan and chitin can be recognized by Dectin-1, CR3, and NOD2, TLR4, respectively.

The *C. albicans* cell wall can be divided into two layers, the outer and inner layers. The outer layer is mainly composed of C-linked glycoproteins such as mannan (80–90%). The inner layer contains chitin, β-1,3-glucan, and β-1,6-glucan (Shibata et al., 2007; Lowman et al., 2014). The host cells can recognize the components of the *C. albicans* cell wall through various pattern recognition receptors (PRR) on their cell surface (Figure 1) (the first step in activating human immunity). Many PRR families, including Toll-like receptors (TLR), C-type lectin receptors (CLR), NOD-like receptors (NLR), and RIG-1-like receptors (RLR), are involved in the fungal recognition process (Jang et al., 2015; Table 1).

**TABLE 1:** Pattern recognition receptors that sense fungal-associated PAMPs.

**Pathogen-recognition receptors (PRRs)**		**Cell type expressing PRRs**	**Pathogen-associated molecular patterns (PAMPs)**
CLRs	DC-SIGN	Macrophage DCs	Mannans
	Dectin1	Macrophage	β-1,3-glucan
	Dectin2	Macrophage Neutrophil DCs	α-mannans
	MINCLE	Monocyte Neutrophil	α-mannosyl residues
	Mannose receptor	Macrophage	N-mannan
TLRs	TLR2	Monocyte	O-mannan
	TLR4	Monocyte	O-mannan
	TLR9	Monocyte Macrophage	Unmethylated DNA
	TLR3	Monocyte Macrophage	Double-stranded RNA
NLRs	NOD2	Monocyte	Chitin
	NLRP3	Monocyte	β-1,3-glucans
Other receptors	Galectin 3	Macrophage	β-mannan
	EphA2	Epithelial cells	β-glucan

### Chitin

Chitin is located in the innermost layer of the *C. albicans* cell wall. Chitin induces interleukin 10 (IL-10) production in neutrophils and macrophages through a nucleotide-binding oligomerization domain with protein 2 (NOD2)-dependent pathway to inhibit host pro-inflammatory response during fungal infection (Davidson et al., 2018; Patricia et al., 2019). I Besides, TLR9 recognizes chitin, which induces several anti-inflammatory cytokines, such as IL-10 (Wagener et al., 2014), that maintain a balanced immune response (Salazar and Brown, 2018).

### Mannan

Mannan and mannoprotein are key components of the *Candida* spp. cell wall and are recognized by various CLRs, including mannose receptor, Dectin-2, dendritic cell (DC) specific ICAM3 capture non-integrin (DC-SIGN), and MINCLE (Cambi et al., 2008; van de Veerdonk et al., 2009; Yamasaki et al., 2009; Saijo et al., 2010). The mannose receptor is found on the macrophage surface and binds to the *Candida* N-mannan (Porcaro et al., 2003; Netea et al., 2008), thus promoting cytokine production, especially IL-17 (van de Veerdonk et al., 2009). Dectin-2 is mainly expressed in DCs, macrophages, and neutrophils, and can recognize *Candida* α-mannan. Dectin-2 also regulates T helper cell 17 (Th 17) response, ROS production, and phagocytosis (Wells et al., 2008; Saijo et al., 2010; Saijo and Iwakura, 2011; Thompson et al., 2019). C-SIGN, expressed in DC cells and macrophages, can recognize N-mannan in *Candida* spp. DC-SIGN activation promotes Th cell activation and differentiation by inducing cytokine production (Ramirez-Ortiz and Means, 2012; Goyal et al., 2018; Speakman et al., 2020). Mannan can also be recognized by TLRs, such as TLR2, TLR4, and TLR6. Furthermore, activation of downstream pathways promotes the expression of pro-inflammatory cytokines during *Candida* infection (Mogensen, 2009).

### β-Glucan

β-glucans, including β-1,3- and β-1,6-glucans, are essential antigen components in the *C. albicans* cell wall (Chaffin et al., 1998). β-glucan is covered by the outermost mannoproteins in the yeast phase, thus preventing *C. albicans* recognition by the body immunity (Garcia-Rubio et al., 2019). The *C. albicans* yeast and hyphae have structurally different β-glucans (Lowman et al., 2014). The *C. albicans* in budding or hyphal forms expose β-glucan during yeast phase to hyphal phase transition (Davis et al., 2014), which the CLR mainly recognizes. Dectin-1 is the most studied β-glucan receptor (Brown et al., 2002; Batbayar et al., 2012). Dectin-1 is expressed primarily on monocytes and macrophages and induces cytokine release and phagocytosis of fungi (Goyal et al., 2018). Dectin-1 also promotes the recognition response of TLR2 and TLR4 (Trinchieri and Sher, 2007; Oliveira-Nascimento et al., 2012), which are associated with cell wall mannan recognition. Dectin-1 signaling pathway can also inhibit the overactivation of neutrophil extracellular traps (NETs) during fungal infections, preventing uncontrolled tissue damage during the immune response (Branzk et al., 2014). β-glucan can also be recognized by complement receptor 3 (CR3), which is essential in the recognition, phagocytosis, and killing of *C. albicans* in neutrophils (van Bruggen et al., 2009; Gazendam et al., 2014).

## Interaction Between *C. albicans* and Host Mucosal Immune Cells

**FIGURE 2:**
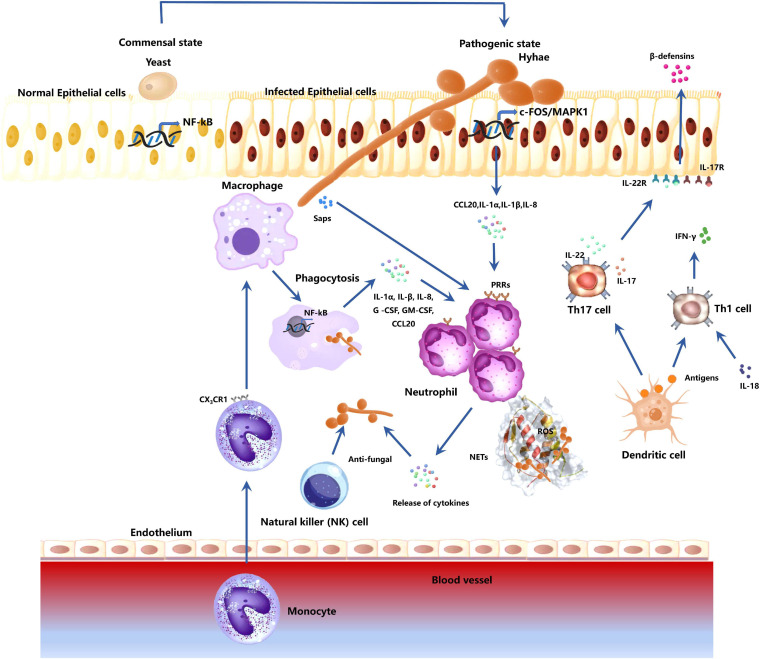
Responses of host immune-related cells during *C. albicans* mucosa invasion. The fungus transforms from a commensal to a pathogenic state by first breaking through the epithelial cells. Epithelial cells activate mitogen-activated protein kinase 1 (MAPK1)- and FOS-related pathways to mediate cytokine production (CCL20, IL-1 α, IL-1 β, IL-8). These cytokines can recruit host immune cells. Epithelial cells also release β-defensins for anti-candida activities. The macrophages in the tissue contribute to the antifungal effect through phagocytosis and secretion of cytokines (IL-1 α, IL-β, IL-8, G-CSF, GM-CSF, CCL20). Monocytes in the blood are recruited. They migrate to the infected sites, differentiate into macrophages, and participates in anti-*C. albicans* process. Neutrophils respond to cytokines secreted by macrophages and epithelial cells, and fungal antigenic substances Saps. Neutrophil activation induces the production of active antifungal substances to kill the fungi. Neutrophil can also release neutrophil extracellular traps (NETs) and reactive oxygen species (ROS) to kill the *C. albicans*, which cannot be engulfed due to the large hyphae. DC cells activate Th cells via antigen presentation. Th17 activation induces IL-17 and IL-22 production, which are involved in the recruitment and activation of neutrophils. IL-17 and IL-22 can also activate epithelial cells and promote the release of -defensins. Activated Th1 cells can secrete IFN-γ, promoting neutrophil and macrophage antifungal effects. NK cells also have a certain resistance to *C. albicans* hyphae.

The oral mucosa structure is similar to that of the skin, composed of the epithelium and lamina propria. The epithelium mainly consists of keratinocytes. The outermost epithelium layer comprises several layers of closely arranged cells known as the stratum corneum, which can be divided into orthokeratosis and parakeratosis. *C. albicans* infection mainly causes epithelial surface edema. Hyphae are found in the outer 1/3 of the keratinized layer or epithelium and are vertically distributed on the epithelial surface, with several neutrophil infiltrations. Hyphae and infiltrated inflammatory cells form microabscesses. Besides, there are several lymphocytes, plasma cells, neutrophils, and other inflammatory cells in the connective tissue below the epithelium. Many immune cells participate in the antifungal process during *C. albicans* infections (Figure 2).

### Epithelial Cells

Epithelial cells are essential in mucosal immunity against fungal invasion. *C. albicans* hyphae invade the epithelial cells via the active penetration and the induced endocytosis processes (Naglik et al., 2011; Mayer et al., 2013). The two processes involve specific pathogen-associated molecular patterns (PAMPs), expressed on the fungal surface, and recognition by pattern recognition receptors (PRRs) of host cells (Mogensen, 2009; Amarante-Mendes et al., 2018). The PRR family members include Toll-like receptors (TLRs), C-type lectin receptors (CLRs), and NOD-like receptors (NLRs) (Netea et al., 2008; Brown, 2011; Gow et al., 2011; Wheeler et al., 2017; Heung, 2020; Pellon et al., 2020; Vendele et al., 2020). Several TLRs are found on the mucosal epithelial cell surface, of which TLR4 directly affects the mucosal defense against *C. albicans* infection (Weindl et al., 2007; Naglik and Moyes, 2011). The epithelial ephyrin type-A receptor 2 (EphA2) was recently identified as a non-traditional PRR involved in *C. albicans* cell wall component and β-glucan identification (Swidergall et al., 2018; Swidergall et al., 2019; Olsen, 2020). This recognition is non-specific, and both *C. albicans* hyphae and yeast can be identified. Besides *C. albicans*, other fungal species can induce EphA2 activation. However, EphA2 activation via *C. albicans* is the most researched (Dambuza and Brown, 2018). A study showed that fungi invasion (oral candidiasis) was significantly high in the EphA2^–/–^ knockout mice with normal immune function than WT mice (Swidergall et al., 2018). Upon detecting abnormal morphology and proliferation of *C. albicans*, the epithelial cells activate the internal mitogen-activated protein kinase 1 (MAPK1) and FOS pathway (Moyes et al., 2010; Moyes et al., 2011). The activated epithelial cells release various pro-inflammatory cytokines and immune cell chemokines to recruit more immune cells to reach the infected area, thus improving immune response. The released cytokines include interleukin-1 α (IL-1 α), interleukin-1 β (IL-1 β), interleukin-8 (IL-8), and chemokine (C-C motif) ligand 20 (CCL20) (Moyes and Naglik, 2011; Swidergall et al., 2018). Epithelial cells can also produce antifungal β-defensins through response activation to IL-22 produced by Th17 cells, inhibiting *C. albicans* invasion (Eyerich et al., 2011; Sparber and LeibundGut-Landmann, 2019; Gaffen and Moutsopoulos, 2020). The latest research shows that IL-22 and IL-17 have a synergistic effect against *C. albicans*. Therefore, IL-22 signaling activation is essential in the oral basal epithelial layer and can cooperate with IL-17 signaling in the oral mucosa (Aggor et al., 2020).

### Macrophages

The macrophages are the key effector cells in the antifungal mucosal defense (Hirayama et al., 2017) and kill *C. albicans* mainly via phagocytosis (Uwamahoro et al., 2014). The effector cells have phagosomes containing enzymes that can produce reactive oxygen species (ROS) and reactive nitrogen species (RNS) (Nüsse, 2011; Uribe-Querol and Rosales, 2017). *C. albicans* are phagocytized into macrophages, where they are constricted in the phagosomes and killed via ROS (Uwamahoro et al., 2014). Macrophages produce chemokines and inflammatory factors, which recruit and activate other immune cells to the infection sites. A study showed that proliferation and lethality of *C. albicans* are significantly higher in macrophages-deficient mice than in normal mice (Qian et al., 1994; Wheeler et al., 2017). Meanwhile, blood monocytes move to the infected site, differentiating into inflammatory macrophages for the anti-infection process (Serbina et al., 2008). The CX_3_C-chemokine receptor 1 (CX_3_CR1) on the macrophage surface is essential in the resistance process to candidiasis (Lee et al., 2018). These results show the role of macrophages in mucosal fungal infection defense.

### Neutrophils

Neutrophils are essential in *Candida* mucosal infection defense. Neutrophils respond to chemokines released by the activated epithelial cells and macrophages, such as IL-1 α, IL-β, IL-8, G -CSF, GM-CSF, CCL20, and β defensin, then moves to the fungal infected tissues (Netea et al., 2008; Moyes et al., 2016; Patin et al., 2019; Pellon et al., 2020). Neutrophils can also directly respond to the *C. albicans* virulence factors, such as the secretory aspartyl proteinases (Saps) (Gabrielli et al., 2016; Singh et al., 2020). Neutrophils are essential in antifungal mucosal immunity. Neutropenia greatly increases the risk of invasive fungal infections (Kullberg et al., 1990; Horn et al., 2009; Hirayama et al., 2020). Neutrophils are the only host cell that can successfully inhibit *C. albicans* transformation from the yeast phase to the hyphae phase (Brown, 2011; Lionakis, 2014). A mouse neutropenia model revealed severe fungal infections (Kullberg et al., 1990). Several PRRs on neutrophil surface, including TLR2, TLR4, TLR9, Dectin-1, Dectin-2, Dectin-3, DC-SIGN, and MINCLE, can recognize *C. albicans* antigenic components (Taylor et al., 2007; Cheng et al., 2012; Zhu et al., 2013; Dühring et al., 2015) thus complete neutrophil activation (Cheng et al., 2012; Kenny et al., 2017; Li et al., 2020). The activated MyD88, inflammatory complex, and SYK can mediate the downstream MAPK and NF-κB signaling pathways in neutrophils, leading to the expression of cytokines and antifungal factors, such as elastase, lactoferrin, β-defensin, lysozyme, gelatinase, and cathepsin G (Amulic et al., 2012; Liew and Kubes, 2019). Neutrophil elastase and cathepsin B also have antifungal activity (McCormick et al., 2010; Shopova et al., 2020). Besides, the phagocytosis and the unique role of neutrophil extracellular traps (NETs) are activated in neutrophils (Cheng et al., 2012; Menegazzi et al., 2012; Kenny et al., 2017). The neutrophils also phagocytize *C. albicans* through the PRRs on the cell surface. For instance, the neutrophils kill *C. albicans* through an oxidative cell reaction (Aratani et al., 2002; Endo et al., 2017) after *C. albicans* recognition via Dectin-1 (Netea et al., 2015). Besides *C. albicans*, Dectin-1 also recognizes β-glucans in the cell wall of many fungal species. The neutrophil cells kill non-phagocytized-*C. albicans* hyphae via NETs (Urban et al., 2006; Menegazzi et al., 2012; Halverson et al., 2015). NETs, formed by several DNA-containing fibril structures, can combine the pathogens and induce the production of antimicrobial substances, such as myeloperoxidase (MPO), lactoferrin, azurocidin, and cathelicidin, for antifungal activity (Urban et al., 2009; McCormick et al., 2010; Papayannopoulos, 2018). Calprotectin is an essential NET (Urban et al., 2009). Protease 3 can hydrolyze cathelicidin to antimicrobial peptide LL-37 (CAMP) (Kościuczuk et al., 2012), which has several antimicrobial effects. CAMP promotes the destruction of the fungal cell membrane by directly binding to the fungus (Zhang et al., 2010; Kahlenberg and Kaplan, 2013), inhibit biofilm formation and fungal adhesion (Tsai et al., 2011), enhancing the production of chemotaxis and ROS, and inhibiting neutrophil apoptosis (Nagaoka et al., 2006; Alalwani et al., 2010). Although both the yeast and hyphal forms of *C. albicans* trigger NETs, neutrophils respond faster to hyphae. Besides killing fungi directly, NETs can slow the hyphae growth, possibly by limiting micronutrients, such as zinc (Urban et al., 2009).

### Natural Killer (NK) Cells

Natural killer cells are essential in innate immunity against fungal invasion (Schmidt et al., 2018). Existing studies mainly focus on the role of NK cells in systemic *Candida* infection, and none has reported its functions in mucosal infections. Studies have shown systemic candidiasis mouse models without NK cells have increased sensitivity to the *Candida* spp. and *Aspergillus* spp. (Whitney et al., 2014; Drummond and Lionakis, 2019). A Similarly, in severe combined immunodeficiency disease (SCID) mice with lymphocyte deficiency, NK cell depletion increases sensitivity to systemic candidiasis (Quintin et al., 2014). NK cells promote immune activation of epithelial cells and phagocytic cells, limiting invasion and spread of *Candida* from the mucosal surface to the deeper layers. Although NK cells cannot inhibit the *Candida* hyphae growth, they significantly affect perforin-dependent antifungal activity (Voigt et al., 2014; Abel et al., 2018). NK cells also have similar roles in *Candida* mucosal infections. However, more evidence is needed to confirm the roles of NK cells in mucosal fungal infection.

### Dendritic Cells (DCs)

The host can produce IFNβ through SYK- and IFN-regulatory factor 5 (IRF5)-dependent pathway, which has an anti-candidiasis effect. Dendritic cells (DCs) are essential during this process (Biondo et al., 2011; del Fresno et al., 2013; Hoepel et al., 2019). DCs mainly recognize antigenic substances in the internal environment and present to T cells (Steinman, 2012). Although DCs are not as effective at *Candida* phagocytosis as macrophages, their antigen presentation role in activating Th cells is crucial (Ramirez-Ortiz and Means, 2012; Richardson and Moyes, 2015).

### T Cells

Many specific T cells are involved in inhibiting *Candida* infections (Verma et al., 2014). Studies have shown that Candida-specific T cells can produce IL-17 and IFN-γ against *Candida* infections (Zielinski et al., 2012; Conti et al., 2016; Verma et al., 2017; McDermott and Klein, 2018). Both Th1 and Th17 are essential in *Candida* infection defense (Conti and Gaffen, 2015; Speakman et al., 2020). Furthermore, Th1 cell response and IFN-γ production are essential during neutrophil and macrophage inhibiting processes against fungal invasion (Netea et al., 2015; Dewi et al., 2017). IL-18 induces Th1 cell activation (Netea et al., 2003; Nakanishi, 2018). A study showed that mice without IFN-γ and IL-18 are more prone to candidiasis. However, IFN-γ or IL-18 treatment reverses the susceptibility (Stuyt et al., 2004). Th17 cells are also essential in the resistance process to *C. albicans* (Hernández-Santos and Gaffen, 2012). Th17 cells produce IL-17 and IL-22, which are involved in the recruitment and activation of neutrophils (Liang et al., 2006). Th17 cells can also activate epithelial cells, which produce β defensins (Eyerich et al., 2011). Recently, studies have proved that Th17 cell response is essential in human anti-mucosal fungal infections (Hernández-Santos and Gaffen, 2012; Mengesha and Conti, 2017). A study showed that mice without IL-17 receptor or downstream signaling elements are more sensitive to oropharyngeal candidiasis (Conti et al., 2016). Several immune cells, including γδ-T cells, NK cells, type 3 innate lymphoid cells (ILC3), and TCRβ + “natural” Th17 cells (nTh17), produce IL-17 (Conti and Gaffen, 2015).

### Functions of Th17/IL-17

The stabilization, degradation, and translation of mRNA are regulated by IL-17, orchestrated by a suite of RNA binding proteins, including Act1. This property of IL-17 explains how it can synergize with a wide range of inflammatory signals. Besides, most of the relevant RNA binding proteins were first identified in studies of IL-17–dependent oral candidiasis (Li et al., 2019). IL-17 can regulate different immune relevant factors, including neutrophil-activating CXC chemokines and G-CSF, antimicrobial β-defensins proteins, cytokines (IL-6 and GM-CSF), and transcription factors (IκBξ, C/EBPβ, and C/EBPδ) (Li et al., 2019). Consistently, IL-17/Th 17 drives potentially damaging inflammation (Stockinger and Omenetti, 2017). Th17 responses are significant in the protection against *C. albicans* (Li et al., 2018). Besides providing protective immunity, Th17 cells contribute to immune pathology. *C. albicans*-specific T cell responses broadly modulate human anti-fungal Th17 immunity by propagating Th17 cells cross-reactive to other fungal species, such as *Aspergillus fumigatus*. This could accentuate acute allergic bronchopulmonary aspergillosis (Bacher et al., 2019). However, IL-17 also drives tissue repair. Barrier tissue repair tends to be the dominant response in the gut (Hueber et al., 2012; Stockinger and Omenetti, 2017). The degree to which IL-17 drives tissue repair in the oral mucosa is poorly understood (Li et al., 2019). Disease-associated fungi trigger IL-6– and IL-23–dependent accumulation of Th17 cells on the oral mucosa. Disease-causing Th17 cells drive tissue damage through excessive neutrophil recruitment and related immunopathology (Dutzan et al., 2017). Meanwhile, *C. albicans* hyphae secrete the peptide candidalysin, which damages oral epithelial cells and triggers the secretion of IL-1 and IL-36. These signals activate innate IL-17–producing cells. IL-17 binds to its receptor on oral epithelial cells and induces the release of antimicrobial effectors, including CXC chemokines and G-CSF. These effectors promote a neutrophil response and direct fungicidal activity (Huppler et al., 2014; Trautwein-Weidner et al., 2015; Conti et al., 2016). Based on the double-edged functions of Th17 cells, which besides protecting barrier tissues, contribute to immunopathology, the importance of Th17/IL-17 in controlling antifungal response remains controversial (Gaffen and Moutsopoulos, 2020).

## *C. albicans* Escape Mechanisms

*C. albicans* has several mechanisms for escaping host immune detection and elimination. For instance, hyphae elongation hinders phagocytic activity or damage phagocytic cells, triggering stress response pathways in fungi and interfering with phagosome maturation.

### Phagocytosis Evasion by Changing Cell Size and Shape

Various phagocytic cells have a conventional cell size, limiting the size of the microorganisms they can engulf. For instance, *C. albicans* has a diameter of between 5 and 10 μm in the yeast phase and over 20 μm in the hyphal phase (Bar-Yosef et al., 2017; Mukaremera et al., 2017), indicating that phagocytosis cannot occur in the latter stage. Conversely, the fungus can perforate the phagocytic cells due to its increased growth rate, thus killing the phagocytic cell (Lewis et al., 2012). Although the RAB protein regulates phagosome maturation in phagocytic cells, *C. albicans* cell wall morphogenesis can prevent phagosome death by interfering with the RAB protein role of phagosome maturation (Alvarez-Dominguez et al., 2008; Kopeckova et al., 2020). Studies have shown that RAB14 localizes to *C. albicans* phagosomes after phagocytosis. RAB14 localization is associated with the morphology of the fungal cells in the phagosome and the size of the hyphae. Loss of RAB14 function delays phagolysosome maturation, increasing *C. albicans* escaping rate and macrophage killing rate (Okai et al., 2015).

### Prevention of Identification and Killing by Changing *C. albicans* Cell Wall Structure

The host enzyme effect and free radical activation trigger fungi cell wall stress sensors. The sensors activate the Mkc1 pathway, leading to Rlm1-dependent activation of chitin which can strengthen cell wall biosynthesis and repair cell wall damages (Fuchs and Mylonakis, 2009; Román et al., 2015). *C. albicans* cell wall composition also affects the function of phagosome-associated RAB protein (Lewis et al., 2012). For instance, mannan loss increases the phagosome function and reduces the ability of fungi to escape the host immune. Mannan has a protective effect on β-glucan, which prevents the exposure and identification of fungal antigens in phagocytic cells (Snarr et al., 2017).

### Prevention of Immune Killing by Activating *C. albicans* Stress Response Pathways

The intracellular phagosomes mainly kill the fungal cells after *C. albicans* endocytosis in phagocytic cells. Phagosomes contain several antimicrobial agents, such as hydrolases and oxidants, which can kill and degrade *C. albicans* cells (Flannagan et al., 2009). Studies have shown that NADPH oxidase activity is associated with fungal oxidative damage, limiting *Candida* cell growth (Brothers et al., 2013; Alves et al., 2020). However, *C. albicans* responds to these stresses mainly through the stress pathways, including mitogen-activated protein kinase (MAPK) Hog1 (Enjalbert et al., 2006), AP1-like transcription factor Cap1, and heat shock transcription factor Hsf1 (da Silva Dantas et al., 2010). ROS and RNS activate the expressions of the transcription factors Cap1 and Cta4, inducing catalase, glutathione, and thioredoxin protective effects. However, removal of nitrous and oxidizing substances in immune cells induces protective effects of flavin hemoglobin Yhb1, thioredoxin, glutathione cycle enzymes glutathione reductase (Glr1), and S-nitrosoglutathione reductase (Fdh3) (Tillmann et al., 2015).

### Host Cell Death Induction

*C. albicans* induces macrophage lysis, especially in the hyphal forms (Schäfer et al., 2014; Okai et al., 2015). *C. albicans* mutants without ergosterol cannot induce macrophage lysis, indicating that specific components of the fungal cell membrane are also necessary for macrophage lysis induction (O’Meara et al., 2015, 2018). Meanwhile, this lysis function is not associated with hyphae formation. Some mutants which can also form the hyphae, such as the *ECE1* mutant, do not lyse macrophages (Kasper et al., 2018). The *ECE1* gene encodes Candidalysin, a major *C. albicans* virulence factor, which damages cells by destroying the host cell membrane. The *ECE1* null mutant also forms hyphae but cannot destroy cells (Moyes et al., 2016). Moreover, ALG1 and ALG11 mutants can also induce macrophage lysis without hyphae formation (O’Meara et al., 2015).

## Others

### Impact of Microbiome on the Outcome of Fungal Infection

The human microbiota consists of bacteria, archaea, viruses, and fungi that build a highly complex network of interactions between each other and the host. *C. albicans*, as a commensal and opportunistic pathogen on the mucosa, often interact with other microbiota and work together to host immunity. *C. albicans*-specific Th17 cells can cross-react with *A. fumigatus* and contribute to pulmonary inflammatory diseases (Bacher et al., 2019). *C. albicans* and *Staphylococcus aureus* have a synergistic effect in mucosal infections (Li et al., 2018), with the former playing a leading role. Invasion and Th17 induction by *C. albicans* and *S. aureus* damage intestinal epithelial cells and release Th17-inducing cytokines (Moyes et al., 2016; Verma et al., 2017). However, some microbes resist *C. albicans* colonization. Commensal anaerobic bacteria, specifically clostridial *Firmicutes* (clusters IV and XIVa) and *Bacteroidetes*, are critical for maintaining *C. albicans* colonization resistance in mice (Fan et al., 2015). Hypoxia-inducible factor-1 α (HIF-1 α), a transcription factor important for activating innate immune effectors, and the antimicrobial peptide LL-37 (CRAMP in mice) are key determinants of this resistance effect (Fan et al., 2015). Understanding how other microbes and fungi interact to influence host health and immune modulation can lead to the development of therapeutic strategies aimed at preventing infection.

### Fungal Pathogens Modify/Interact With Epithelial and Immune Cells

The host immune attack modifies itself in various ways to destroy fungal pathogens. Immune cells secreted proteins such as complement bind to fungal wall components, such as β-1,6-glucan, resulting in enhanced phagocytosis (Rubin-Bejerano et al., 2007). In *C. albicans*, mannan protects β-glucan, preventing its exposure and identification. Host-derived immune cells release lytic enzymes to destroy the integrity and architecture of the fungal cell wall (Wheeler et al., 2008; Wagener et al., 2014). For different forms of fungi, host immune cells have devised specific response strategies to chemotactic signals released by hyphae (Jouault et al., 1998) while neutrophils migrate more rapidly toward yeast cells (Rudkin et al., 2013). This often leads to an increase in macrophage death (Rudkin et al., 2013). Besides, host phagocytes can exist with *C. albicans* without killing them or being killed by fungal lytic mechanisms (Bain et al., 2012). In mouse macrophages, actin and phosphoinositides are dynamically recruited to fully formed phagosomes containing *C. albicans* to prevent fungal escape (Heinsbroek et al., 2009). Although this mechanism can benefit host immune cells in avoiding lysis and death, it allows the fungus to spread to uninfected areas (Casadevall, 2010). Epithelial cells reply to *C. albicans* and candidalysin by activating epidermal growth factor receptor (EGFR) (Moyes et al., 2014). Inhibition of EGFR strongly suppresses candidalysin-induced MAPK signaling (c-Fos/MKP1) and GM-CSF and G-CSF release (Ho et al., 2019; Naglik et al., 2019). This impairs neutrophil recruitment (Liles et al., 1997; Gaviria et al., 1999) and amplifies *C. albicans* infections (Ho et al., 2019).

## Outlook

The microbial composition of the human mucosa is diverse and structurally complex. As the first line of defense for human immunity, the mucosa interacts with the microorganisms on its surface to keep the host healthy. However, *C. albicans* is the most prevalent fungus on the mucosa surface and causes numerous fungal diseases. The incidence of *C. albicans* infections has gradually increased due to the high occurrence of systemic diseases, such as tumors, Acquired Immune Deficiency Syndrome (AIDS), liver and kidney disorders, the widespread development of interventional therapy, organ transplantation, and the abuse of various antibiotics. The interaction between mucosal immunity and *C. albicans* involves many interconnected mechanisms, which can provide new drug candidate targets against *C. albicans* infection. The host immune regulation mechanism provides a basis for developing compounds that can activate specific host defenses, thus maximizing the killing of *C. albicans* and minimizing the damage to normal host cells. Furthermore, the self-protection mechanisms of *C. albicans* against the host immunity provide further information on how to effectively block the immune escape of *C. albicans.*

## Author Contributions

YZ, LC, YL, XZ, and BR: conception and design of the work and drafting the work. YZ, LC, and BR: revised the manuscript. XZ and BR: final approval of the manuscript to be published. XZ, YL, and BR: agreement to be accountable for all aspects of the work. All authors contributed to the article and approved the submitted version.

## Conflict of Interest

The authors declare that the research was conducted in the absence of any commercial or financial relationships that could be construed as a potential conflict of interest.
